# Worldwide Occurrence of HIV-Associated Neurocognitive Disorders and Its Associated Factors: A Systematic Review and Meta-Analysis

**DOI:** 10.3389/fpsyt.2022.814362

**Published:** 2022-05-31

**Authors:** Yosef Zenebe, Mogesie Necho, Wondwosen Yimam, Baye Akele

**Affiliations:** ^1^Department of Psychiatry, College of Medicine and Health Sciences, Wollo University, Dessie, Ethiopia; ^2^Department of Nursing, College of Medicine and Health Sciences, Wollo University, Dessie, Ethiopia; ^3^Department of Pharmacy, College of Medicine and Health Sciences, Wollo University, Dessie, Ethiopia

**Keywords:** meta-analysis, HIV/AIDS, hand, world, wide

## Abstract

**Background:**

HIV-associated neurocognitive disorders are common in people living with HIV/AIDS and affect the adherence of patients to prescriptions, activities of daily living, and quality of life of patients. However, there is a lack of summative evidence in the area. The present meta-analysis was therefore addressing this gap.

**Methods:**

We did our electronic search in Psych-Info, EMBASE, Scopus, and PubMed. The retrieved articles were stored with the endnote reference manager and data was extracted using Meta-XL version 5.3. The quality of studies was evaluated with the modified Newcastle–Ottawa Scale (NOS). A random-effect model and STATA-16 were used to compute the average estimate of HAND. Heterogeneity was weighed with I^2^ statistics. A sensitivity analysis and subgroup analysis were employed. The existence/nonexistence of a publication bias was checked with the Eggers test of publication bias.

**Results:**

The average prevalence of HAND was 50.41% (95% CI: 45.56, 55.26). The average estimate of HAND in Europe was found to be 50.015% whereas in Africa, Asia, and the United States of America (USA) it was 49.566, 52.032, and 50.407% respectively. The prevalence of HAND in studies that used the HIV Dementia Scale (IHDS) was 36.883% and 59.956% at cutoff points of IHDS <9.5 and IHDS <10 respectively. Besides, the estimated average of HAND with the global dementia scale (GDS) was 40.766%. The prevalence of HAND in cross-sectional, cohort, and case-control studies was 49.52, 54.087, and 44.45% in that order. Socio-demographic variables; low level of education and older age, clinical and HIV related variables; the advanced stage of the illness and CD4 count of 500 cells/dl or less and psychological variables such as comorbidity of depression increases the risk of HAND.

**Conclusion:**

The prevalence of HIV-associated neurocognitive disorders was about 50.41%. Low level of education and older age, clinical and HIV related variables such as the advanced stage of the illness and CD4 count of 500 cells/dl or less, and comorbidity of depression were associated with HIV associated neurocognitive disorders. Public health interventions for HIV patients should target these essential problems.

## Introduction

HIV/AIDS is a global public health issue with more than 34 million people living with HIV/AIDS(PLHIV) currently (1). Mental, neurological, and substance (MNS) related disorders are very common in People living with HIV/AIDS (2). The latest systematic review and Meta-analysis studies by Necho et al. (3) revealed that 35.8% of HIV/AIDS patients had depressive symptoms (3). Another systematic review and meta-analysis studies reported that the prevalence of post-traumatic stress disorder (PTSD), alcohol use disorder (AUD), and suicidal ideation in individuals living with HIV/AIDS were 32.67% (4), 22.02% (5), and 21.7% (6), respectively.

Since HIV is a neurotropic virus, it affects the cortical and subcortical parts of the brain resulting in cognitive impairment (7). This impact of HIV on the cognitive domain of patients is known as HIV-associated neurocognitive disorder (HAND) (8, 9). The level of HAND ranges from asymptomatic impairment to minor neurocognitive disorder and full-blown dementia (10–13). The HIV-associated neurocognitive disorder affects memory, attention, problem-solving ability, language, higher executive function, and independent activities of daily living (14).

HIV-associated neurocognitive disorders are very common in HIV/AIDS patients (15). A study by Habib et al. (16) reported that the burden of neurocognitive impairment (NCI) among patients on Antiretroviral therapy (ART) attendants was 30.39%. Based on the report of multiple earlier studies the worldwide burden of HIV-associated neurocognitive disorders (HAND) varies from a minimum of 7.3% to a maximum of 85% (8, 10, 12–14, 17–50). Besides, the frequency of HIV-associated neurocognitive disorder (HAND) in developed and developing countries varies between 19–52% (31, 51), and 14–64% (12, 13), respectively.

Different studies reported varieties of socio-demographic and clinical factors associated with HIV-associated neurocognitive disorders in people living with HIV/AIDS. For example studies from, Cameroon, Nigeria, Botswana, Singapore, Malawi, and Dessie Ethiopia reported that socio-demographic variables such as older age, female sex, and lower educational level were risk factors for HIV associated neurocognitive disorder (13, 14, 46, 50, 52, 53). Besides, from Clinical variables CD4 count of <500 cells/mm3 was related to HIV-associated neurocognitive disorder based on reports of studies from Brazil, Singapore, and Northern Nigeria (14, 50, 54). Moreover, advanced stages of AIDS and not being on highly active antiretroviral treatment (HAART) were associated with HIV-associated neurocognitive disorder in South Africa (51–53). In Uganda, behavioral and psychological variables such as depression, Body mass index, and alcohol abuse were associated with HIV-associated neurocognitive disorder (10). Moreover, medication non-adherence and opportunistic infections were associated with HIV-associated neurocognitive disorder (46, 55).

The presence of HIV-associated neurocognitive disorder predisposes people living with HIV/AIDS to substance abuse, poor medication adherence, and unsafe sex so the poor quality of life and loss of follow-up from treatment are outcomes. These conditions speed up the progression of the virus to its advanced stages and the development of severe opportunistic infections and death (11, 12).

Even though a high proportion of the world population has been living with HIV/AIDS and a high prevalence of mental, neurological, and substance use disorders in this population, these problems, especially neurocognitive disorders, are not investigated well. Despite the presence of some studies in the area, they are mostly confined to a small population and a narrow geographical area (8, 10, 12–14, 17–50). Consequently, there arises a need to have aggregate data regarding HIV-associated neurocognitive disorder and its associated factors from the global context.

Therefore, this systematic review and meta-analysis aimed to estimate the prevalence of HIV-associated neurocognitive disorder in people living with HIV AIDS and to analyze the associated factors for HIV-associated neurocognitive disorder in people with HIV AIDS.

## Methods

### Search Strategy

We have performed our search strategy for this review in different ways. Initially, we did an electronic exploration for eligible articles regarding HIV-associated neurocognitive disorders in people living with HIV AIDS in the databases of Psych-Info, EMBASE, Scopus, and PubMed. As a sample of our search strategy with the PubMed database, we have used the following key terms: **[(neurocognitive disorder or HIV-associated neurocognitive disorder or HAND) and (adults)] and (Human immunodeficiency virus or HIV or acquired immunodeficiency syndrome or AIDS)**. Moreover, Psych-Info, EMBASE, and Scopus databases were investigated in line with the searching guidelines of each database. Besides, the reference lists of included studies were searched manually for additional eligible articles. There was no time restriction to the publication year of the articles during the searching process.

### Eligibility Criteria

During our study of a systematic review and meta-analysis on HIV-associated neurocognitive disorder in people living with HIV AIDS, we have set the following inclusion and exclusion criteria based on the: 1) the primary inclusion criteria to analyze where the study should assess prevalence OR associated factors of HIV-associated neurocognitive disorder in people living with HIV AIDS. 2) The HIV-associated neurocognitive disorder had also to be investigated using the International HIV Dementia Scale (IHDS), Frascati criteria, Mini-mental state exam(MMSE), global dementia scale(GDS), Brief Neurocognitive Screen, Neuropsychological battery, Montreal Cognitive Assessment (MoCA), In-depth neuropsychological assessment, Wechsler Adult Intelligence Scale and ADC.

We excluded studies (1) that assessed neurocognitive disorder in samples other than people living with HIV/AIDS. (2) That assessed neurocognitive disorder in individuals taking psychotropic medication. (3) Studies that are letters to the editor with non-original data content, earlier reviews, case studies, studies involving non-human subjects, and articles published in a language other than the English language were also excluded from the analysis. After all relevant articles were searched in the mentioned databases; they were stored in an endnote reference manager. Two of the authors (MN and YZ) individually screened the titles and abstracts of articles stored in an endnote reference manager using the eligibility criteria. Next to that, the above two authors carefully read the full length of articles that passed the initial screening and decided independently articles suitable for inclusion in the final meta-analysis. Any disagreement between them regarding eligibility criteria was resolved by agreement and with a third reviewer (WY).

### Data Extraction and Quality Assessment Techniques

The extraction of relevant data from the 40 final included articles was separately done by two authors (MN and YZ) using an identical data extraction form as suggested by PRISMA guidelines (56), using Meta-XL version 5.3 (57) and the result was summarized in a table. Disagreements among these two authors were settled with a discussion. The contents of the data extraction template were author name, year of publication, the country where the study was done, study design, study sample population, an assessment tool for HIV associated neurocognitive disorders, number of cases with HIV associated neurocognitive disorders, prevalence of HIV associated neurocognitive disorders, sampling technique employed to recruit participants, and response rate of the study.

The quality of 40 included studies (8, 10, 12–14, 17–50) had been evaluated using the modified Newcastle–Ottawa Scale (NOS) (58) as the gold standard. Representativeness of sample and sample size, statistical quality, comparability among participants, and ascertainment of cases were the components of this quality assessment scale. Based on this scale studies with a quality score of 7 to 10 were categorized as very good/good, a score of 5 to 6 was categorized as having satisfactory quality, and a score less than 5 was taken as unsatisfactory quality.

### Data Analysis and Synthesis

The random-effect model was used to compute the average estimate of HIV-associated neurocognitive disorders and their associated factors with 95% CIs (59). The STATA-16 Meta-prop package (60) was employed to find the average estimate of HIV-associated neurocognitive disorders. Heterogeneity among the 40 involved studies (8, 10, 12–14, 17–50) was weighed with *Q* and *I*^2^ statistics (61). An *I*^2^ numerical value of more than 50% implies a significant degree of heterogeneity among 40 studies (61). As there existed a potential heterogeneity during analysis, we further conducted a sensitivity analysis to identify an influential study outweighing the study found. Additionally, we did a subgroup analysis regarding the country of the study, study design, and the assessment tools used to screen HIV-associated neurocognitive disorders. The presence/absence of a publication bias was done with the funnel plot test (62) and eggers test of publication bias.

## Results

### Identification of Studies

**Figure 1:**
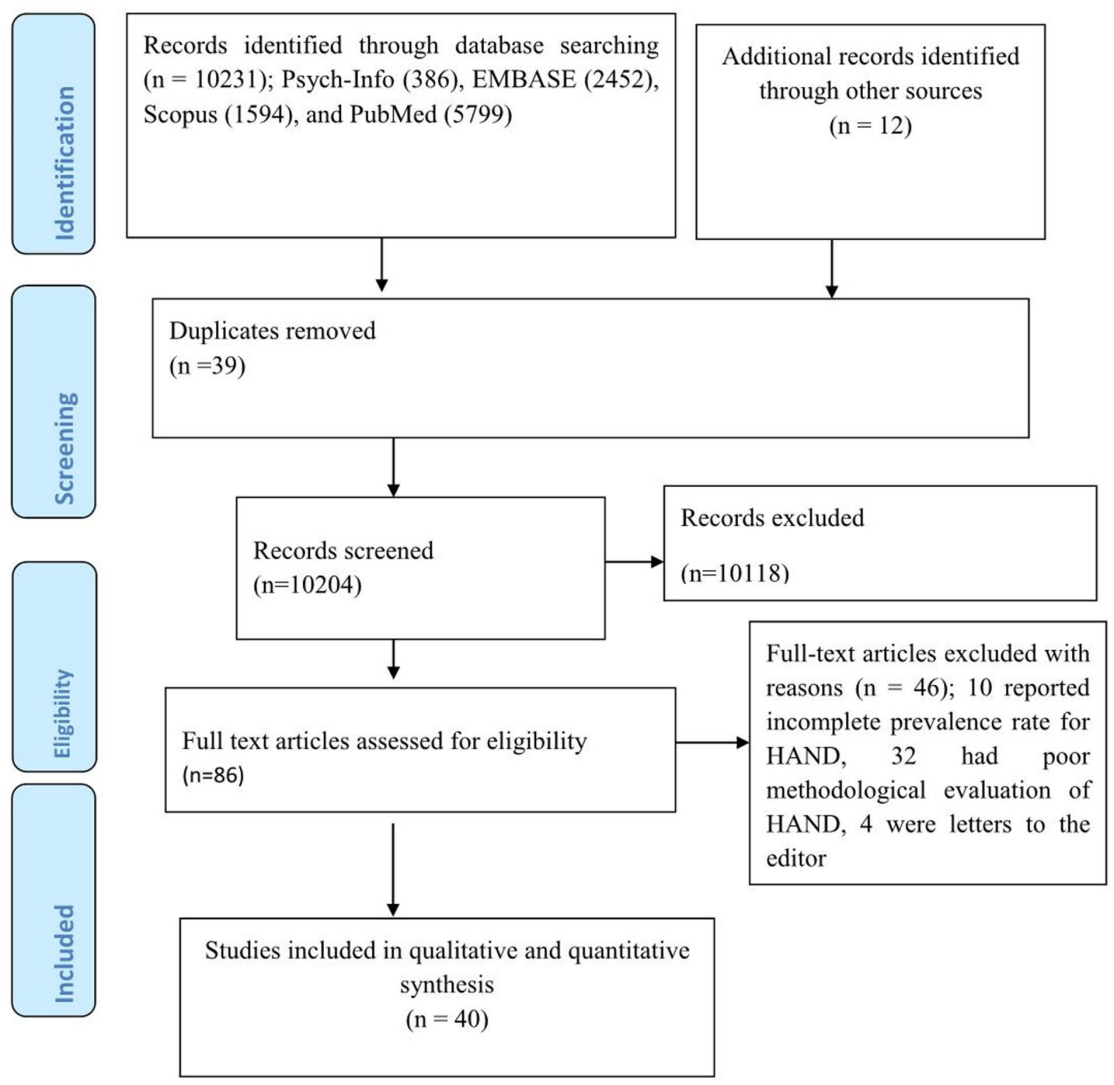
PRISMA flow chart for the review search process.

Our electronic search gave a total of 10231 articles; Psych-Info (386), EMBASE (2,452), Scopus (1,594), and PubMed (5,799). Additionally, 12 articles were retrieved by looking for a reference list of earlier articles. Thus, a total of 10,243 articles were retrieved during the overall searching process, of which 39 were removed as they were duplicates. During the initial stage of screening, most of the articles (10,118) were excluded merely by looking at their title or abstract. The remaining 86 articles were completely inspected for suitability for inclusion in the study but only 40 articles were suited for the final meta-analysis as the remaining 46 studies were excluded; 10 reported an incomplete prevalence rate for HAND, 32 had a poor methodological evaluation of HAND, and 4 were letters to the editor (Figure 1).

### Characteristics of Included Studies

A total of 40 studies (8, 10, 12–14, 17–50) that surveyed HIV-associated neurocognitive disorders in 14,107 HIV/AIDS patients were integrated into the current systematic review and meta-analysis study. Of the 40 included studies; 11 were from Europe, 21 were from Africa, six were from Asia, and two were from the United States of America (USA). Most of the included studies were cross-sectional in design whereas the remaining 10 and 2 were cohort and case-control, respectively. Regarding tools used for the assessment of HIV-associated neurocognitive disorders, half of the included studies (twenty) used the International HIV Dementia Scale (IHDS). Frascati criteria, global dementia scales (GDS), and Montreal Cognitive Assessment (MoCA) were also used to assess HIV-associated neurocognitive disorders in three, three, and three studies, respectively. The reported prevalence of HIV-associated neurocognitive disorders included in the meta-analysis differs from 7.3% in the United Kingdom (28) to 88% in Kenya (35) (Table 1).

**Table 1:** Characteristics of studies on HIV associated neurocognitive disorders in HIV/AIDS patients which are incorporated in this meta-analysis.

**References**	**Country**	**Study design**	**Sample size**	**Tools with cut off points**	**Sampling technique**	**Response rate**	**Age of respondents**	**Prevalence of HAND**	**Cases with the outcome**	**CD4 count**	**Viral load**	**Study population**
Lawler et al. (13)	Botswana	CS	120	IHDS ≤ 9.5	Randomly selected	100%	M & F 21-50 years	39.2%	47	20%, CD4 count <200/mm3	80%, <400 copies/ml	HIV positive individuals
Pinheiro et al. (14)	Brazil	CS	434	IHDS ≤ 10	NA	90.3%	M & F ≥18 years	HAND =54.1%	235	14.4%, CD4 count <200/mm3	6.3%, <50 copies/ml	HIV positive individuals
Elham et al. (26)	Iran	**CS**	93	Frascati neuropsychological criteria	NA	100%	M & F 18**–**60 years	HAND=50.5%	47	Mean CD4 count is 536.47(254.4)		On ART patients
Haddow et al. (28)	UK	CS	150	ADC	Randomly selected		M & F Median age = 43 years	HAND =7.3%	11	Median, 540 cells/mL		HIV positive people
Kelly et al. (31)	Malawi	CS	106	Frascati criteria	Consecutively	93.8%	M & F >18 years	HAND =70%	74	Median CD4 count 323.5		On ART patients
Yakasai et al. (47)	Nigeria	CS	80	Frascati criteria	NA	100%	≥18 years	HAND=40%	32			Both ART& ART naïve patients
Belete et al. (20)	Ethiopia	CS	254	IHDS ≤ 9.5	Systematic random sampling technique	92.1%.	M & F 18-64 years	HAND=33.3%	85	10.7%, CD4 count <200/mm3		HIV positive people
Araya et al. (18)	Ethiopia	CS	584	Mini-mental state exam	Systematic random sampling	99.49%	≥18 years	HAND=35.6%	208			
Yitbarek et al. (49)	Ethiopia	CS	328	IHDS	Systematic random sampling	97.04%	≥18 years	HAND=37.7%	124			
Belete et al. (20)	Ethiopia	CS	423	IHDS	Systematic random sampling	100%	≥18 years	HAND=24.8%	105			
Nyamayaro et al. (38)	Zimbabwe	CS	155	GDS ≥0.5	NA	100%	M & F 18 years or older	HAND=49.7%	77	Median (range) CD4 count 520 (300-699)		On ART patients
Tsegaw et al. (46)	Ethiopia	CS	595	International HIV Dementia Scale (IHDS) ≤ 9.5	Systematic random sampling technique	99%	M & F 18 and 65 years	HAND=36.4%	217	60.9%, CD4 count <500/mm3		On ART patients
Focà et al. (27)	Italy	cohort	206	MMSE		100%	>18 years	HAND= 47.1%	97			
Pascal et al. (39)	Central African Republic	CS	244	International HIV Dementia Scale (IHDS) ≤ 8.36		100%	M & F >18 Years	HAND= 25%	61	Average CD4 was 175 ± 126 CD4/mm3		On ART patients
Awori et al. (19)	Kenya	CS	218	MoCA <26.	Consecutively sampled	98.6%	18 – 65 years	HAND = 69%	150			HIV positive people
Achappa et al. (17)	India	CS	101	IHDS ≤ 10	Convenient sampling	100%	M & F 18-60 years	HAND=90.1%	91	Mean CD4, 450.9 ± 283.49		On ART patients
Sunmonu et al. (45)	Nigeria	Prospective	58	WAIS		100%	M & F >16 years	HAND=63.8%	37	None has CD4 count <200/mm3		HIV positive people
Robertson et al. (40)	Europe and Canada	CS	2,884	Brief Neurocognitive Screen		99.3%	M & F ≥18 years	HAND=41.5%	1,197			Both ART& ART naïve patients
Chan et al. (9)	Singapore	CS	132	MoCA		100%	M & F 21 to 80 years	HAND =22.7%	30			
Cysique et al. (24)	China	Cohort	192	Neuropsychological battery		94.6%	Mean (SD)= 40.2 (6.3)	HAND = 27%		Median rangeCD4 count 375 (11–1,173)		HIV positive people
Harezlak et al. (29)	USA	cohort	268	ADC stage ≥ 1		89.6%	Median=47.0 (43.0–57.0)	HAND = 48%	129			On ART patients
Nakasujja et al. (37)	Uganda	CS	156	IHDS	Consecutively recruited	100%	M & F 18–59 years	HAND =64.7%	101			HIV positive people
Robertson et al. (41)	USA	cohort	1,160	Brief Neuro- Cognitive Screen	Randomized Trials	100%	M & F 34-55 years	HAND=65%	754	Median(range) CD4 count 424 (438-408)		On ART patients
Chan et al. (23)	Singapore	Cohort	53	(MoCA)= ≥ 26 MMSE IHDS ≤ 10		100%	Males >21 years	HAND=52.8%	28			HIV positive people
Kabuba et al. (30)	Zambia	C-C	266	GDS ≥ 0.5		100%	M & F 18 to 65 years	HAND=34.6 %	93	Mean CD4 count/SD 480.28 (242.60)	80.6%, undetectable viral load	On ART patients
Yechoor et al. (63)	Uganda	CS	181	GDS≥ 0.5		100%	M & F 18–50 years	HAND =38%	69			On ART patients
Nakku et al. (10)	Uganda	CS	680	International HIV Dementia Scale (IHDS)≤10		90.9%	M & F ≥18 years	HAND=64.4%.	438		Undetectable VL, 66.6% (n=76)	HIV positive people
Troncoso et al. (8)	Brazil	CS	114	International HIV Dementia Scale (IHDS) ≤ 10		97.4%	M & F ≥18 years	HAND =53.2%	61	7.9%, CD4 count <200/mm3	1.8%,VL ≥100,000 copies/ml	On ART patients
Fasel et al. (64)	Switzerland	Cohort	30	In-depth neuropsychological assessment		100%	M & F ≥18 years	HAND=83%	25	Median CD4 count, 658 cells/μL (IQR 497–814)		HIV positive people
Oshinaike et al. (65)	Nigeria	CC	208	IHDS ≤ 10 MMSE=26	Consecutively	100%	M & F 18-60 years	HAND=54.3%	113	Mean CD4 count/SD 257.2		On ART patients
Atashili et al. (12)	Cameroon	CS	400	International HIV Dementia Scale (IHDS) ≤10	Consecutively	100%	M & F 18 to 55 years	HAND =85%	340			On ART patients
Bonnet et al. (21)	France	Cohort	400	Neurocognitive tests	Consecutively	100%	M & F ≥18 years	HAND =58.5%	234	Median CD4 cell count was 515 cells/ml		On ART patients
Simioni et al. (44)	Belgium	Cohort	200	IHDS ≤10			M & F, Median age of 46.	HAND= 84%	168			
Saini et al. (43)	India	cohort	80	IHDS ≤10	Randomly selected	100%	21 to 50 years	HAND=32.50%	29			On ART patients
Marin-Webb et al. (33)	Germany	Cohort	480	International HIV Dementia Scale (IHDS) ≤ 10			M & F 19 to 80 years	HAND=43%	207	Median CD4 cell count was 554cells/ml		On ART patients
Yusuf et al. (50)	Nigeria	CS	418	IHDS ≤ 9.5		100%	M & F ≥18 years	HAND =21.5%	90			On ART patients
McNamara et al. (34)	Ireland	CS	604	Weschler Adult Intelligence Scale		100%	M & F >18 years	HAND =51.5%	311	Mean/SD CD4 cell count was 538/259.16cells/ml		Both ART& ART naïve patients
Debalkie Animut et al. (25)	Ethiopia	CS	684	International HIV Dementia Scale (IHDS) <9.5	Systematic random sampling method	98%	M & F 18 to 64 years	HAND =67.1%	459	Mean CD4 count was 610 ± 278 cells/mm3		On ART patients
Muniyandi et al. (36)	India	**CS**	33	IHDS ≤10	Consecutively	100%	M & F 25 to 50 years	HAND =63.6%	21			
Mugendi et al. (35)	Kenya	CS	345	International HIV Dementia Scale (IHDS) ≤10 MOCA≤26	Convenient sample	100%	M & F Mean age=42 years (SD ± 9.5)	HAND =88%	304	Median CD4 count, 446 cells/ mm3 (IQR) 278–596		On ART patients

*ANI, Asymptomatic neurocognitive impairment; CC, case control; CD, Cognitive decline; CS, cross-sectional; F, female; GDS, global dementia scale; HAD, HIV associated dementia; HAND, HIV associated neurocognitive disorders; IHDS, International HIV Dementia Scale; II, Intellectual impairment; M, male; MMSE, Mini-mental state exam; MND, Mild neurocognitive disorders; MoCA, Montreal Cognitive Assessment; NA, Not available; NCI, Neurocognitive impairment; SNI, Symptomatic neurocognitive impairment; UK, United kingdom; USA, united states of America*.

### Quality of Included Studies

Among the 40 included studies; the majority (twenty-nine) had scored from 7 to 10 so good quality scores on the scale. Of the remaining 11 studies, seven had a satisfactory quality, and the remaining four studies had unsatisfactory quality (Additional File 1).

### The Prevalence of HIV-Associated Neurocognitive Disorders Among People Living With HIV/AIDS

**Figure 2:**
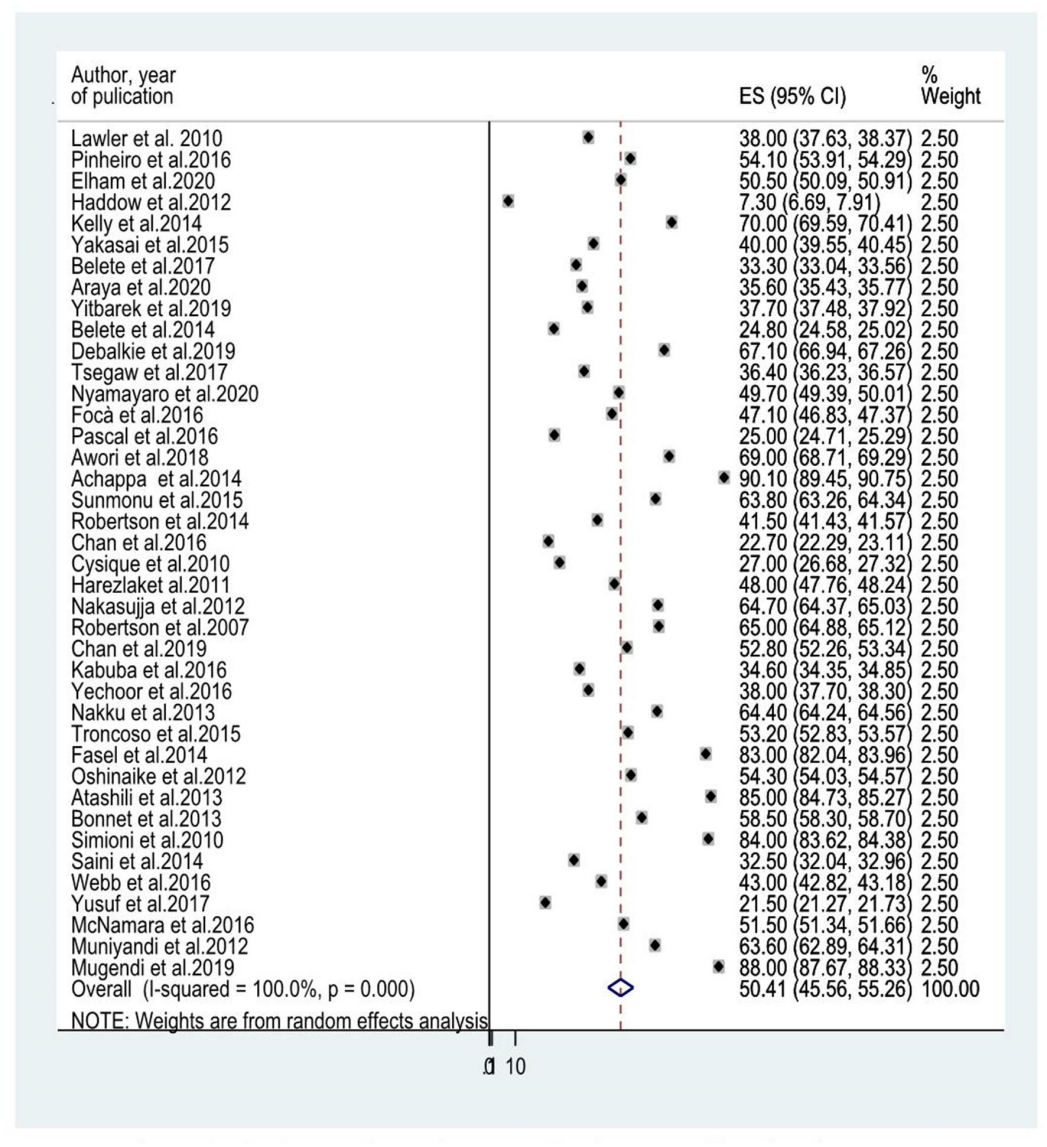
A forest plot for the prevalence od HIV associated neurocognitive disorders.

Forty studies that evaluated HIV-associated neurocognitive disorders in HIV/AIDS had been included to determine the average prevalence of HIV-associated neurocognitive disorders. The reported prevalence of HIV-associated neurocognitive disorders included in the meta-analysis differs from 7.3% in the United Kingdom (28) to 88% in Kenya (35). The average prevalence of HIV-associated neurocognitive disorders using the random effect model was 50.41% (95% CI: 45.56, 55.26) (*I*^2^ =100%, *p* ≤ 0.001; Figure 2).

### Subgroup Analysis of the Prevalence of HIV Associated Neurocognitive Disorders Among People Living With HIV/AIDS

**Figure 3:**
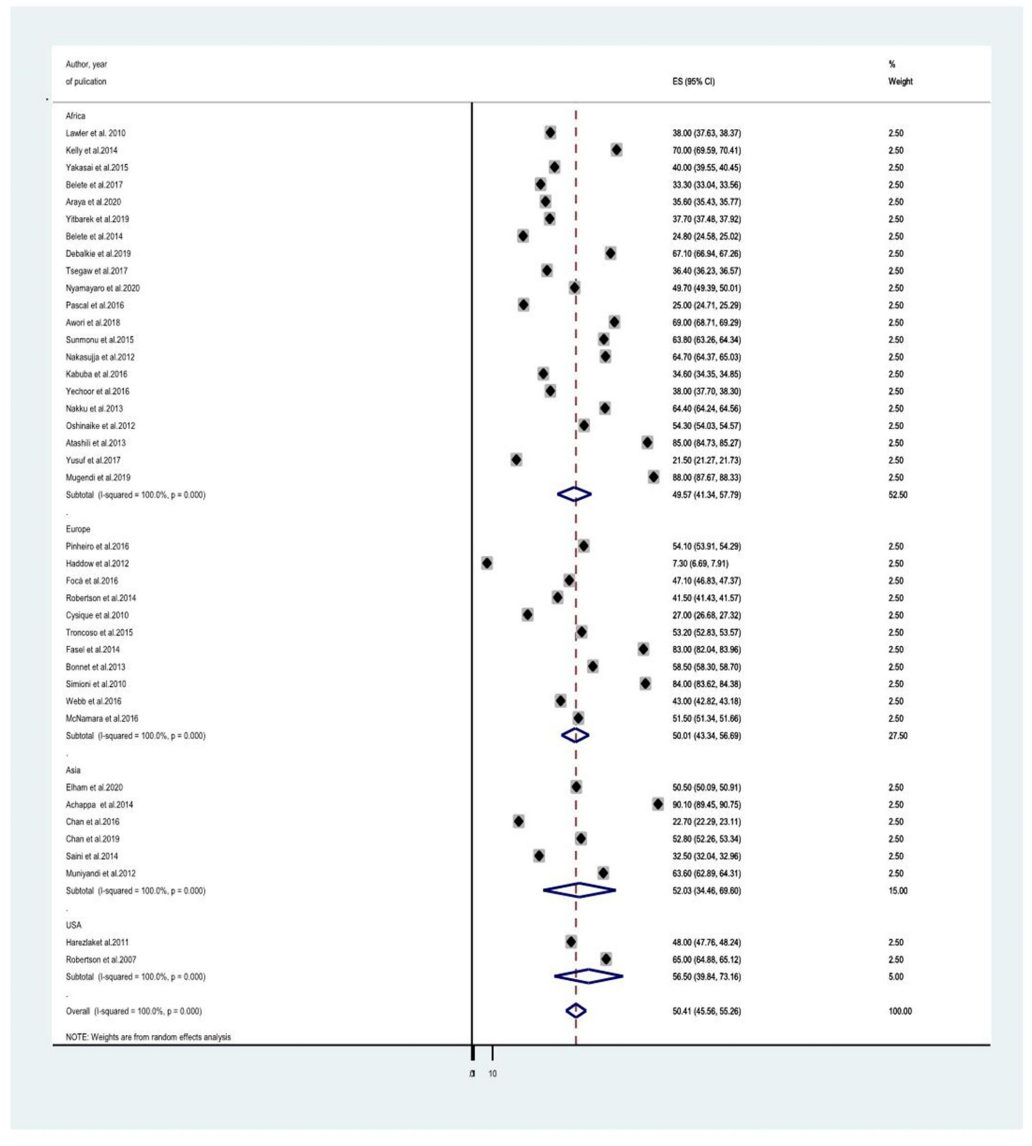
A subgroup analysis for the prevalence of HIV associated neurocognitive disorders based on country of study origin.

**Figure 4:**
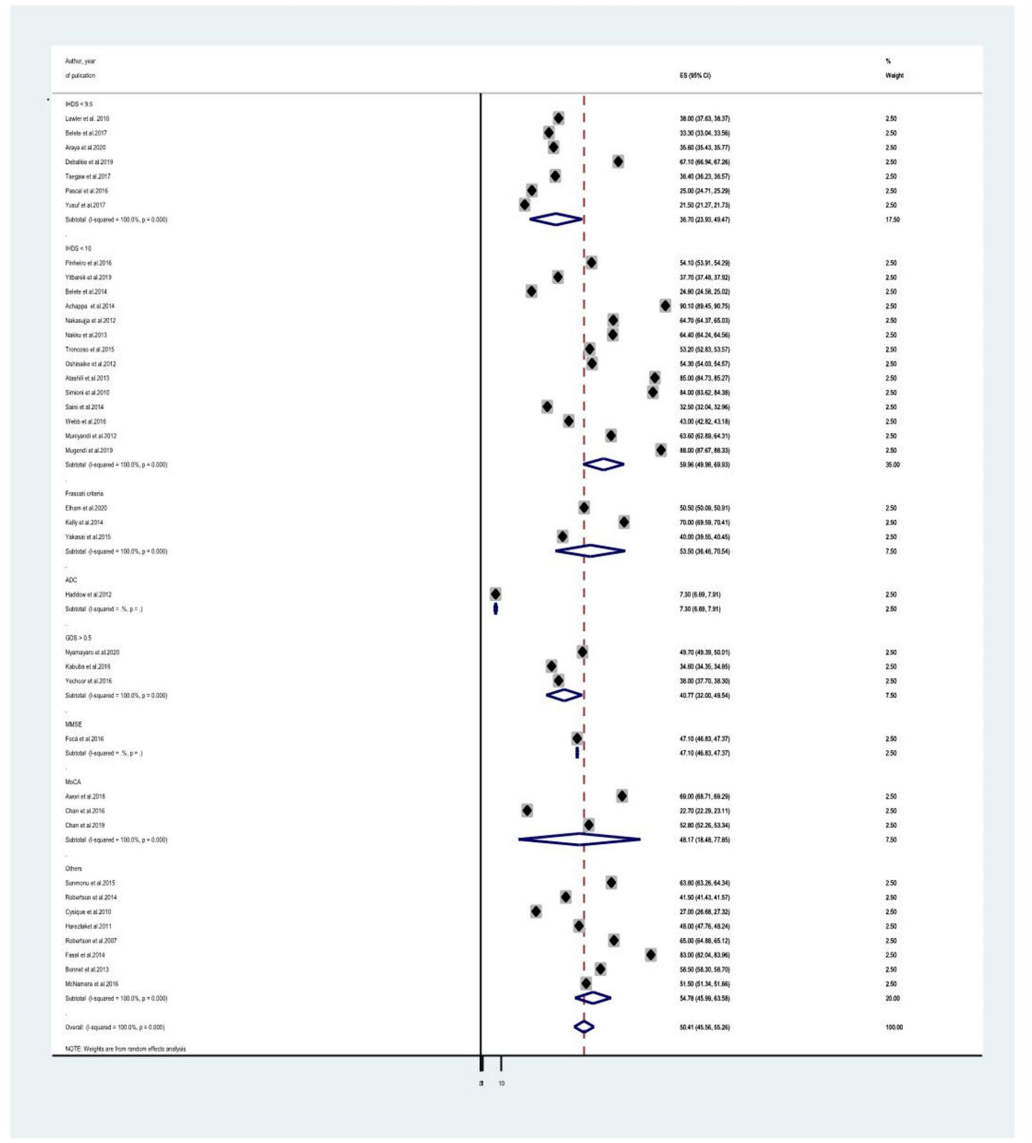
A subgroup analysis for the prevalence of HIV associated neurocognitive disorders based on study tools.

**Figure 5:**
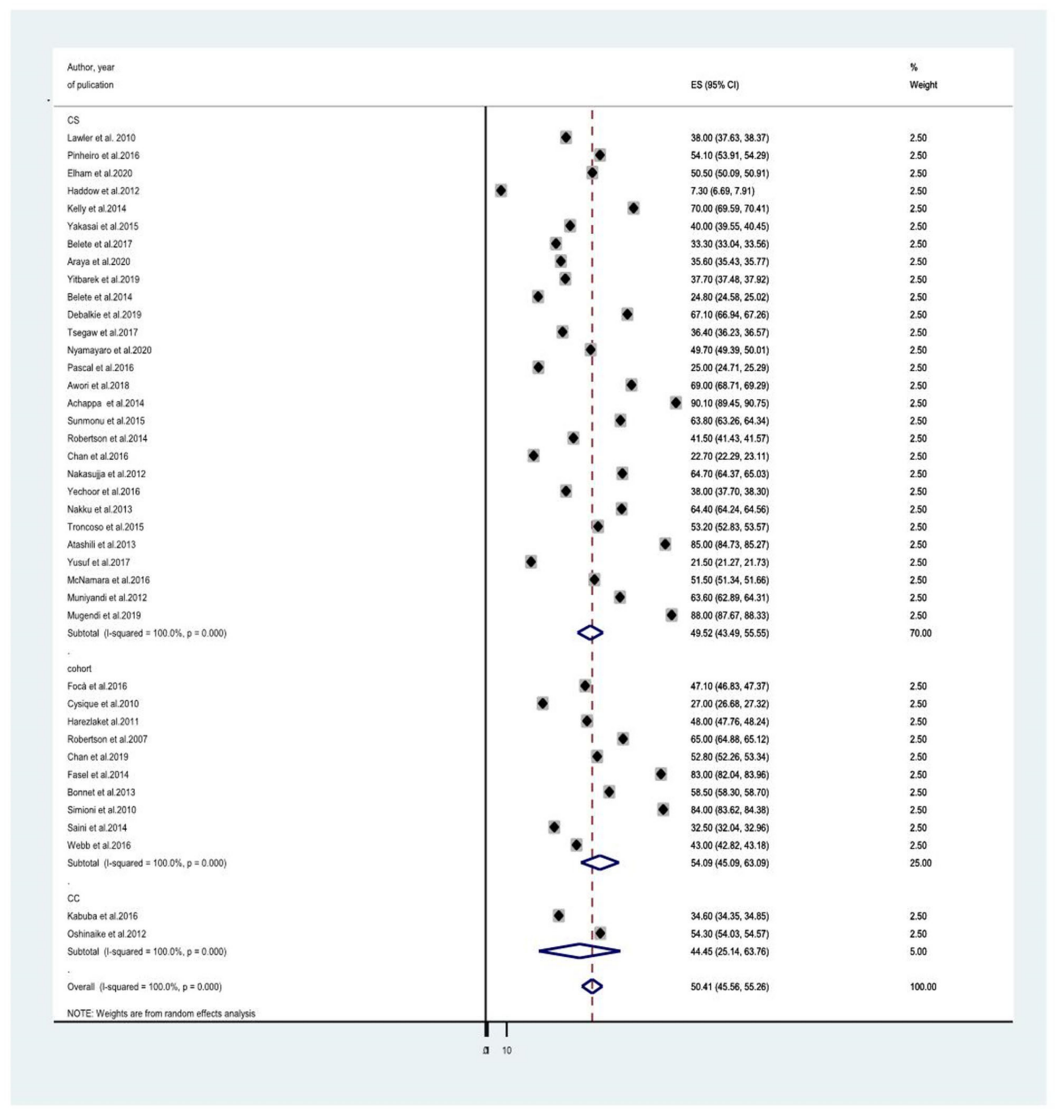
A subgroup analysis for the prevalence of HIV associated neurocognitive disorders based on country of study tools.

Since the average estimate of HIV-associated neurocognitive disorders was predisposed to considerable heterogeneity, we employed a subgroup analysis based on the country where the study was done, the assessment tool used to screen HIV-associated neurocognitive disorders, and the study design. The average estimate of HIV associated neurocognitive disorders in Europe (8, 14, 21, 23, 24, 27, 28, 40, 41, 44) was found to be 50.015% (95% CI: 43.339, 56.691) whereas in Africa (10, 13, 18, 20, 25, 30, 31, 35, 37–39, 45–50), Asia (17, 26, 34, 36, 43) and the United States of America (USA) (29, 41) the average prevalence of HAND were 49.566% (95% CI: 41.342, 57.791) with (*I*^2^ = 96.6%, *p* < 0.001), 52.032 % (95% CI: 34.46, 69.604) with (*I*^2^ = 98%, *p* < 0.001) and 50.407% (95%CI: 45.555, 55.258) (*I*^2^ =100%, *P* < 0.001), respectively (Figure 3 and Table 2). The average estimate of HIV associated neurocognitive disorders in studies which used International HIV Dementia Scale (IHDS) (8, 10, 12–14, 17, 20, 25, 35–37, 39, 43, 44, 46, 48–50, 66) was 36.883% (95%CI: 21.196, 52.571) and 59.956% (95%CI: 49.985, 69.928) at a cutoff points of IHDS <9.5 and IHDS <10, respectively (Figure 4). The estimated average of HAND in studies used the global dementia scale (GDS) (30, 38) was 40.766% (95%CI: 31.995, 49.537). The estimated average of HAND in cross-sectional (8, 10, 12–14, 17–20, 23, 25, 26, 28, 30, 31, 34–40, 45–50) cohort (8, 21, 23, 24, 27, 29, 41, 43, 44) and case-control (30, 66) studies was 49.52% (95% CI: 43.490, 55.545) (*I*^2^ = 48.6%, *P* =1.00), 54.087% (95% CI: 45.087, 63.087) (*I*^2^ = 96%%, *P* < 0.001) and 44.45% (95% CI: 25.144, 63.756) (*I*^2^ = 94.8%, *P* < 0.001), respectively (Figure 5).

**Table 2:** A sensitivity analysis of the prevalence of HIV associated neurocognitive disorders in HIV/AIDS patients when each indicated studies are omitted at a time with its 95% confidence interval.

**S.No**	**Study omitted**	**Estimated prevalence of HAND**	**[95% Conf. Interval]**
1	Lawler et al. (13)	48.55587	48.520576,48.591164
2	Pinheiro et al. (14)	48.256584	48.220829, 48.292343
3	Elham et al. (26)	48.443535	48.408272, 48.478798
4	Haddow et al. (28)	48.594143	48.558952,48.629333
5	Kelly et al. (31)	48.303158	48.267899, 48.338417
6	Yakasai et al. (47)	48.511402	48.476162, 48.546646
7	Belete et al. (20)	48.739456	48.703999, 48.77491
8	Araya et al. (18)	49.037079	49.001163, 49.07299
9	Yitbarek et al. (49)	48.732273	48.696697, 48.767849
10	Belete et al. (20)	49.074566	49.038979, 49.110153
11	Debalkie et al. (25)	47.50845	47.472431, 47.544464
12	Tsegaw et al. (26)	49.017803	48.981865, 49.053738
13	Nyamayaro et al. (38)	48.478603	48.407894, 48.443249
14	Focà et al. (27)	48.481682	48.446255, 48.517109
15	Pascal et al. (39)	48.808884	48.773487, 48.844276
16	Awori et al. (19)	48.145374	48.109974, 48.180775
17	Achappa et al. (17)	48.337997	48.302814, 48.37318
18	Sunmonu et al. (45)	48.392574	48.357368, 48.427784
19	Robertson et al. (40)	50.478935	50.439026, 50.518841
20	Chan et al. (23)	48.652199	48.616936, 48.687466
21	Cysique et al. (24)	48.723553	48.688202, 48.7589
22	Harezlak et al. (29)	48.468979	48.43346, 48.504494
23	Nakasujja et al. (37)	48.270924	48.235588, 48.306259
24	Robertson et al. (41)	46.92638	46.889656, 46.963104
25	Chan et al. (9)	48.440395	48.40519, 48.475601
26	Kabuba et al. (30)	48.73357	48.698093, 48.769051
27	Yechoor et al. (63)	48.604374	48.568996, 48.639748
28	Nakku et al. (10)	47.618401	47.582355, 47.654449
29	Troncoso et al. (8)	48.415298	48.380005, 48.450592
30	Fasel et al. (64)	48.41259	48.377434, 48.447746
31	Oshinaike et al. (65)	48.360401	48.324974, 48.395828
32	Atashili et al. (12)	47.850132	47.814709, 47.885555
33	Bonnet et al. (21)	48.135502	48.099808, 48.171196
34	Simioni et al. (44)	48.149261	48.113976, 48.184547
35	Saini et al. (43)	48.55426	48.519024, 48.589497
36	Marin-Webb et al. (33)	48.673512	48.637695, 48.709328
37	Yusuf et al. (50)	49.084835	49.049297, 49.120373
38	McNamara et al. (37)	48.30397	48.267956, 48.339989
39	Muniyandi et al. (36)	48.421654	48.386478, 48.456829
40	Mugendi et al. (35)	47.994511	47.959171, 48.02985

*HAND, HIV associated neurocognitive disorders*.

### Sensitivity Analysis

In addition to subgroup analysis, we did a sensitivity analysis to know whether one or more of the individual studies outweighed the overall estimate of HIV-associated neurocognitive disorders. The result however reported that the average estimate of HIV-associated neurocognitive disorders ranges from 46.92638% (95% CI: 46.889656, 46.963104) to 50.478935% (95% CI: 50.439026, 50.518841) when each study was omitted from the analysis (Table 2). This implies that there was no single influential study outweighing the average estimate.

### Publication Bias

**Figure 6:**
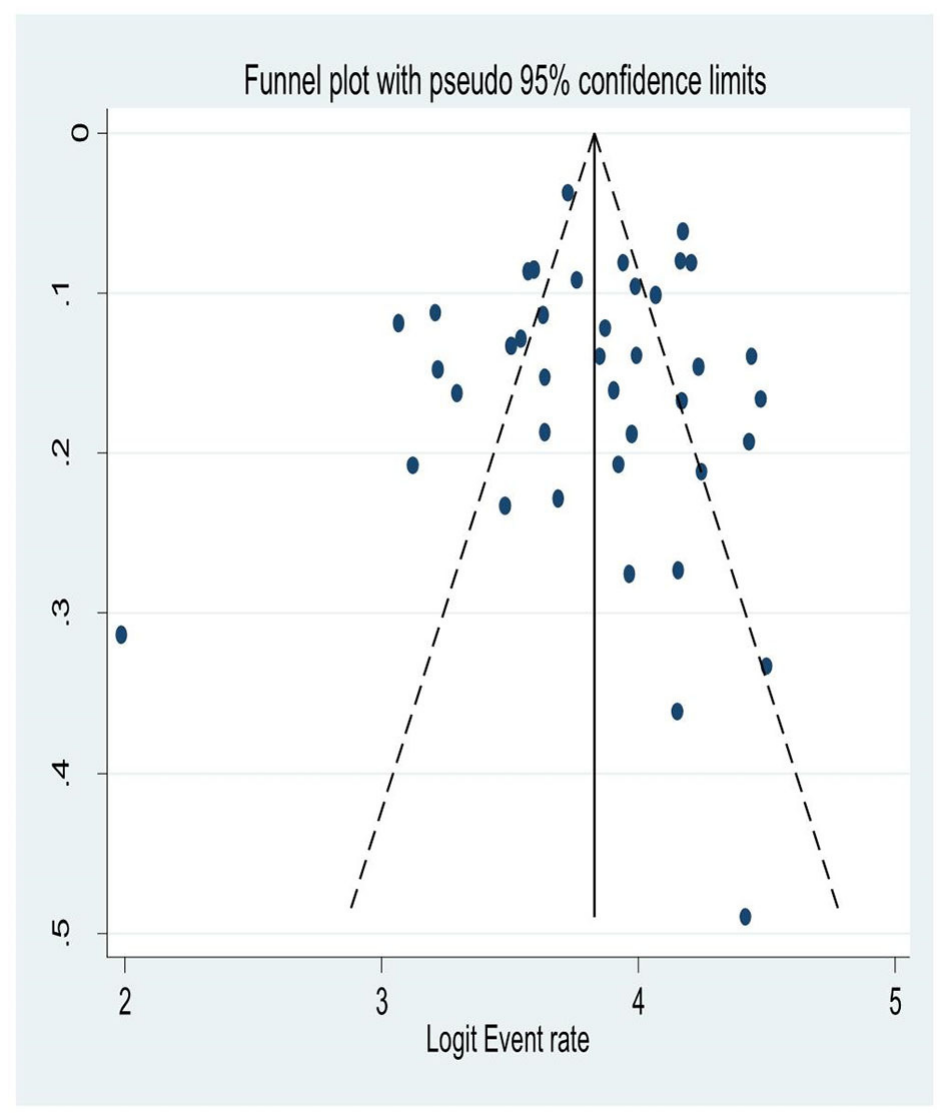
A funnel plot for the prevalence of HIV associated neurocognitive disorders.

Although a graphical inspection from a funnel plot for a Logit event rate of occurrence of HIV-associated neurocognitive disorders in people living with HIV/AIDS alongside its standard error suggests asymmetrical distribution, the quantitative Eggers test of publication bias had been run and its *p*-value was not significant; (*P* = 0.55). This suggests there was no publication bias for the prevalence HIV associated neurocognitive disorders (Figure 6).

### Associated Factors of HIV Associated Neurocognitive Disorders Among People Living With HIV/AIDS

Among the 40 studies, only 15 studies described the factors related to HIV-associated neurocognitive disorders (8, 10, 12, 14, 18, 20, 21, 25, 26, 34, 35, 46–49). The most frequently reported sociodemographic variable as the associated factor of HIV-associated neurocognitive disorders were the low level of education (12, 14, 18, 21, 30, 46, 47) and older age (8, 14, 20, 46, 49). Among clinical and HIV-related variables late clinical stage of the illness (20, 21, 25, 49) and a CD4 count of 500 cells/dl or less (8, 18, 46) were the most commonly described factor for HIV associated neurocognitive disorders. Besides, from psychological variables comorbidity of depression increases the risk of HIV-associated neurocognitive disorders (14, 21, 35). Moreover, clinical and HIV related variables such as impairment in the activity of daily living (20), duration of HIV infection > 5 years (26), poor medication adherence (46), co-morbid medical illness, highest prior VL >100,000 copies/ml (8), history of neurological disease (21), body mass index< 16 kg/m^2^ (25), plasma HIV-1 RNA load between 1.7log10 and 3log10 copies/ml (49), having a co-morbid opportunistic infection (20) and psychological variables like negative life events, high-stress score index (score>10) (10) were related to HIV associated neurocognitive disorders (Table 3).

**Table 3:** Characteristics of associated factors for HIV associated neurocognitive disorders in HIV/AIDS patients by their Odds ratio, Confidence interval, association strength, author and year of publication.

**Associated factors**	**Odds ratio(AOR)**	**95% CI**	**Strength of association**	**References**
Age of 50 years and older	4.85	2.34, 10.03	Strong and positive	Pinheiro et al. (14)
Less than eight years of education	6.72	3.98, 11.32	Strong and positive	Pinheiro et al. (14)
Non-white skin color	1.71	1.04, 2.83	Moderate and positive	Pinheiro et al. (14)
Depression	1.96	1.12, 3.42	Moderate and positive	Pinheiro et al. (14)
Duration of HIV infection > 5 years	3.1	1.70, 7.40	Strong and positive	Elham et a.l (26)
Low level of education	1.2	1.04, 1.44	Weak and positive	Yakasai et al. (47)
Late clinical stage of the illness	4.2	1.19,14.44	Strong and positive	Belete et al. (20)
Impairment in the activity of daily living	7.19	1.73, 21.83	Strong and positive	Belete et al. (20)
CD4 count of 500 cells/dl or less	2.368	1.524, 3.680	Moderate and positive	Tsegaw et al. (46)
No formal education	4.287	2.619, 7.016	Strong and positive	Tsegaw et al. (46)
Poor medication adherence	1.487	1.010, 2.180	Weak and positive	Tsegaw et al. (46)
Older age	3.309	1.259, 8.701	Strong and positive	Tsegaw et al. (46)
6 to 10 Negative life events	2.14	1.45, 3.15	Moderate and positive	Nakku et al. (10)
11 ad more Negative life events	2.35	1.33,4.13	Moderate and positive	Nakku et al. (10)
Medium Stress Score index (score 1–10)	2.55	1.73, 3.77	Moderate and positive	Nakku et al. (10)
High Stress Score index (score >10)	3.29	1.99, 5.45	Strong and positive	Nakku et al. (10)
Female gender	2.66	1.22, 5.82	Moderate and positive	Troncoso and Conterno (8)
Older age	2.87	1.24, 6.64	Moderate and positive	Troncoso and Conterno (8)
Co-morbid medical illness	2.56	1.17, 5.55	Moderate and positive	Troncoso and Conterno (8)
CD4 count <200 cell/mm3	2.71	1.25, 5.86	Moderate and positive	Troncoso and Conterno (8)
Highest prior VL >100,000 copies/ml	2.62	1.12, 6.16	Moderate and positive	Troncoso and Conterno (8)
Low level of education	8.33	3.85, 16.67	Strong and positive	Atashili et al. (12)
Having HIV symptoms	12.16	3.08, 48.05	Strong and positive	Atashili et al. (12)
Advanced AIDS stage	4.87	1.59, 14.90	Strong and positive	Bonnet et al. (21)
Techniqual school level of education	2.16	1.31,3.55	Moderate and positive	Bonnet et al. (21)
Lower than diploma level of education	3.39	1.48, 7.80	Strong and positive	Bonnet et al. (21)
Generalized anxiety symptoms	2.99	1.67, 5.14	Strong and positive	Bonnet et al. (21)
Depression symptoms	2.11	1.23, 3.63	Moderate and positive	Bonnet et al. (21)
History of neurological disease	2.05	1.18, 3.58	Moderate and positive	Bonnet et al. (21)
African country of birth	11.075	4.94, 24.84	Strong and positive	McNamara et al. (34)
Use of benzodiazepines	6.746	2.37, 19.18	Strong and positive	McNamara et al. (34)
Unemployed	2.16	1.2, 3.84	Moderate and positive	McNamara et al. (34)
Body mass index <16 kg/m^2^	4.39	1.60, 12.02	Strong and positive	Debalkie Animut et al. (25)
Unemployed status of occupation	3.18	1.752, 5.777	Strong and positive	Debalkie Animut et al. (25)
Advanced stage of AIDS	3.56	1.406–9.006	Strong and positive	Debalkie Animut et al. (25)
Depression	7.47	1.69, 43.53	Strong and positive	Mugendi et al. (35)
Female gender	2.17	1.02, 4.71	Moderate and positive	Mugendi et al. (35)
Older age	3.1	1.3, 7.4	Strong and positive	Yideg Yitbarek et al. (48)
Plasma HIV-1 RNA load between 1.7log10 and 3log10 copies/ml	2.2	1.1, 4.3	Moderate and positive	Yideg Yitbarek et al. (48)
Plasma HIV-1 RNA load ≥ 3log10 copies/ml	7.5	2.6, 21.5	Strong and positive	Yideg Yitbarek et al. (48)
Khat chewing	4.4	2.3, 8.3	Strong and positive	Yideg Yitbarek et al. (48)
Advanced stage of AIDS	5.6	1.7, 19.2	Strong and positive	Yideg Yitbarek et al. (48)
Having no education	3.11	1.37, 7.04	Strong and positive	Mossie et al. (55)
Older age	4.25	1.05, 17.18	Strong and positive	Mossie et al. (55)
Having co morbid opportunistic infection	7.48	4.1, 13.64	Strong and positive	Mossie et al. (55)
Substance use	4.64	2.3, 9.36	Strong and positive	Mossie et al. (55)
Having no education	5.16	2.20, 12.07	Strong and positive	Araya et al. (18)
Primary education	3.29	1.46, 7.29	Strong and positive	Araya et al. (18)
Having a CD4 count (cells/μl) ≤ 500	1.61	1.11, 2.39	Moderate and positive	Araya et al. (18)
Lifetime use of tobacco	2.4	1.44, 4.01	Moderate and positive	Araya et al. (18)

*AIDS, Acquired Immune deficiency Syndrome*.

#### Association Between Old Age and HIV Associated Neurocognitive Disorders Among People Living With HIV/AIDS

**Figure 7:**
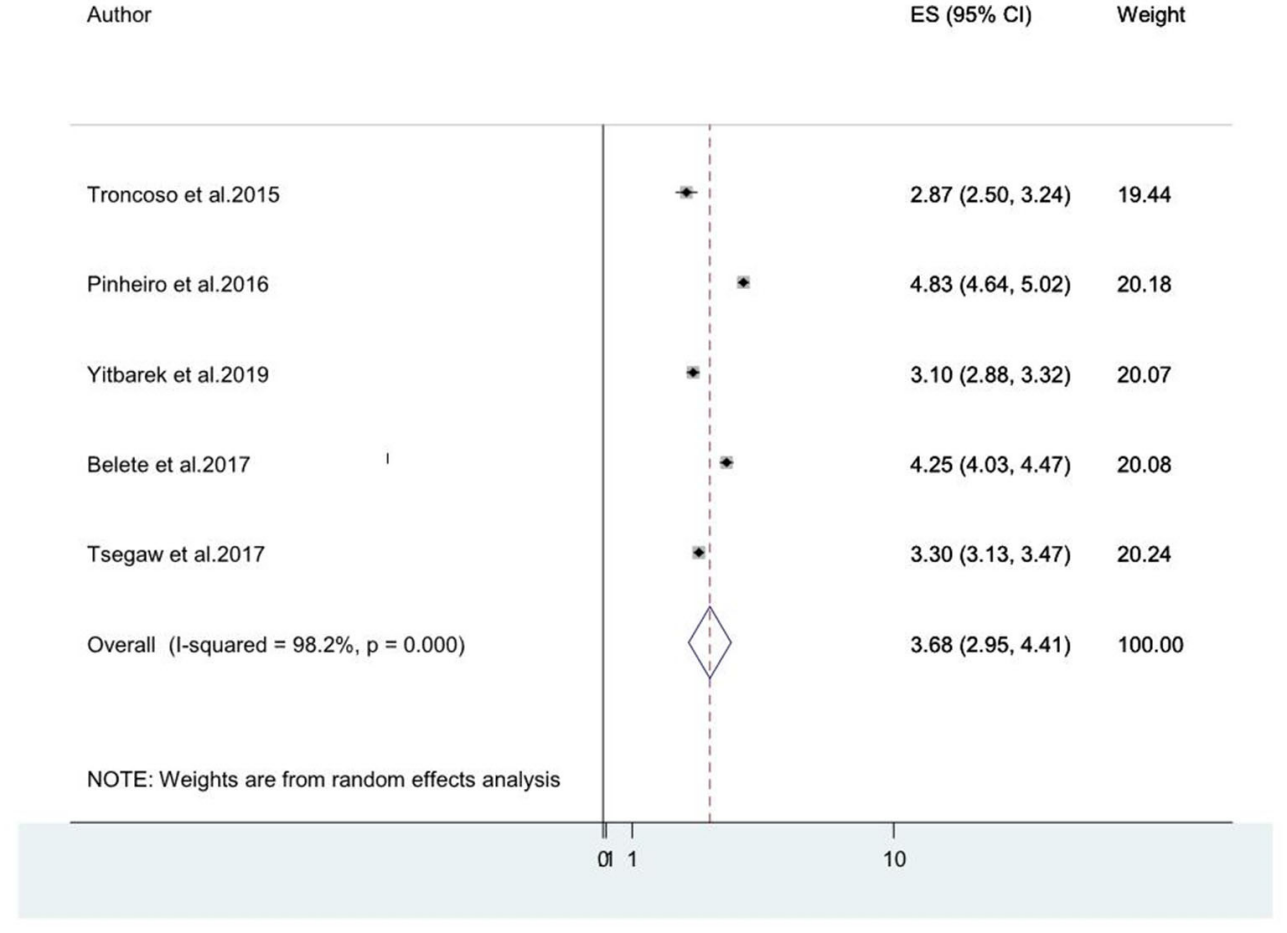
A forest plot for the pooled odds ratio of associated between old age and HIV-associated neurocognitive disorder.

Older age was reported as the risk factor for HIV-associated neurocognitive disorders by five studies (8, 14, 20, 46, 49). The pooled odds ratio for the association between old age and HIV-associated neurocognitive disorder among these five studies was found to be 3.68 (95% CI: 2.95, 4.11) (*I*^2^ = 98.2%%, *P* = 0.000; Figure 7).

#### Association Between Depression and HIV Associated Neurocognitive Disorders Among People Living With HIV/AIDS

As reported in three studies (14, 21, 35) that assessed HIV-associated neurocognitive disorders, depression increases the risk of HIV-associated neurocognitive disorders. The pooled odds ratio for the association between depression and HIV-associated neurocognitive disorder among these three studies was found to be 2.87 (95% CI: 0.87, 4.87) (*I*^2^ = 99.6%%, *P* = 0.000).

#### Association Between Advanced Stages of AIDS and HIV Associated Neurocognitive Disorders Among People Living With HIV/AIDS

**Figure 8:**
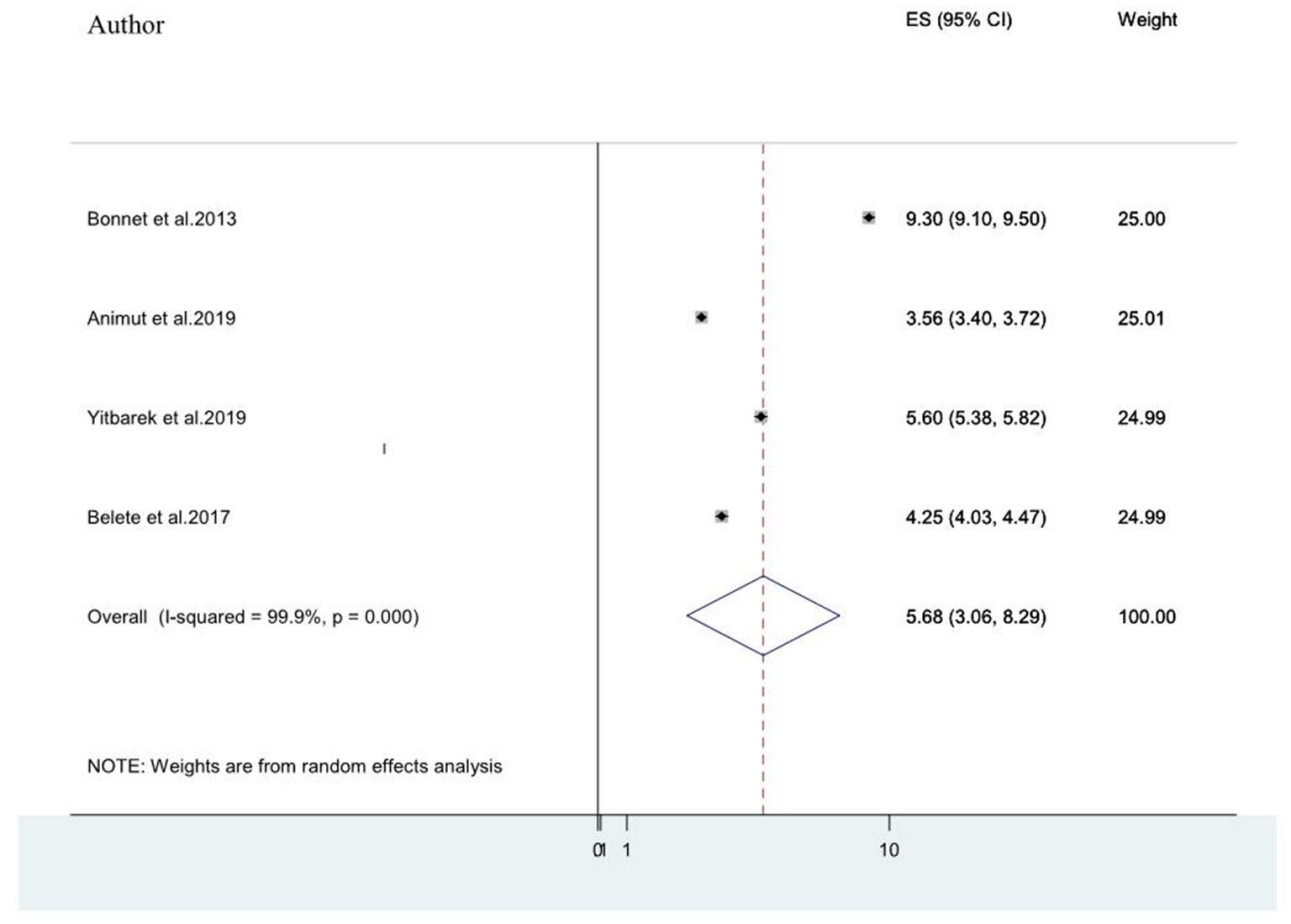
A forest plot for the pooled odds ratio of associated between advanced stage of AIDS and HIV-associated neurocognitive disorder.

Advanced clinical stages of the illness (stage III and stage IV AIDS) (20, 21, 25, 49) were also associated factors for HIV-associated neurocognitive disorders. The pooled odds ratio for the association between advanced stage of AIDs(stage III& IV) and HIV-associated neurocognitive disorder among the four included studies was found to be 5.68 (95% CI: 3.06, 8.29) (*I*^2^ = 99.9%%, *P*< 0.001; Figure 8).

#### Meta-Regression

Firstly, we performed a univariate regression analysis to select the independent variables to incorporate in the final meta-regression model. Then, all variables with *P* < 0.8 were included in the final regression analysis as recommended by Ferrari et al. 2013 (67). The impacts of the country of origin of the study (Europe, Asia, and Africa), Assessment Tool (IHDS < 9.5, IHDS < 10, Frascati criteria, ADC, MMSE, GDS > 0.5, MoCA, and Other Tools), and study design (case-control, cohort and cross-sectional) were quantified in the meta-regression model. The analysis was conducted for the overall effect on the burden of HIV-associated neurocognitive disorders in HIV/AIDS patients. The overall proportion of variance explained by these covariates in the final model was 11% (*R*^2^ = 11%; *P* = 0.302). All the three covariates such as country of origin of the study, Assessment Tool, and study design were not statistically significant determinants for the observed variation in the association between HIV-associated neurocognitive disorders and HIV/AIDS.

## Discussion

To our knowledge, this is the first systematic review and meta-analysis that assessed the global burden of HIV-associated neurocognitive disorders in HIV/AIDS patients. So, the data synthesized will be an important suggestion to varied stakeholders. Overall, 40 studies (8, 10, 12–14, 17–50) that measured the prevalence of HIV associated neurocognitive disorders in 14,107 participants from over 28 different countries and 15 studies that described the factors related HIV associated neurocognitive disorders (8, 10, 12, 14, 18, 20, 21, 25, 26, 34, 35, 46–49) were included.

The result of the present study advocated that more than half of people living with HIV/AIDS were affected with HIV-associated neurocognitive disorders. This suggests that HIV-associated neurocognitive disorders are an important public health problem in people living with HIV/AIDS. The result of HIV-associated neurocognitive disorders is consistent with the prevalence of neurocognitive disorders in patients with chronic kidney disease and diabetes; 48% In the UK (68). However, it was higher than the magnitude of neurocognitive disorders in the general urban population in India (1.5%) (69), and the prevalence of dementia in an urban Turkish population (20%) (70). It was also higher than the prevalence of dementia in Norwegian elders of age 75 and above where dementia was prevalent in 16.3% of the population (71). Besides, the present finding was higher than the prevalence rate of dementia among japans community aged 65 or older; 6.7% (72). The added presence of opportunistic central nervous disease (73–75) in HIV-positive people could be attributing factor to this.

Among studies included in the current review and meta-analysis, the epidemiologic data regarding the prevalence of HIV associated neurocognitive disorders showed a substantial variation across the measurement instrument for HIV associated neurocognitive disorders, and the nature of the study design; however comparable HAND results were found across European, Africa, Asian, US countries. Of the 40 included studies, 20—the majority—were from Africa (10, 13, 18, 20, 25, 30, 31, 35, 37–39, 45–50).

The current study result was higher than the result of a meta-analysis that assessed 16 studies in sub-Sahara Africa where the prevalence of HAND was 30.39% (16). The difference in the study population and significant variance in the number of included studies might cause the variation. Moreover, the risk factors for HIV-associated neurocognitive disorders might be different in Sub-Saharan Africa and the world in general, which may cause the difference in HIV-associated neurocognitive disorders. On the other hand, the current study was higher than the result of the prevalence of Frascati-Criteria-Based HIV-Associated Neurocognitive Disorder; 43.9% (76). The possible reason for this could be due to the difference in sensitivity and specificity between Frascati-Criteria and other tools to screen HIV-Associated Neurocognitive Disorder.

In a subgroup analysis by type of measurement instrument used for HAND, we found that the prevalence was higher as measured with IHDS < 10 (8, 10, 12–14, 17, 20, 25, 35–37, 39, 43, 44, 46, 48–50, 66) (59.956%) than the result measured with Frascati criteria (53.5%), ADC (27.65%), MMSE (41.349%), and GDS (40.766%) with all these differences at statistically significant level(P < 0.001). The possible differences in the estimated magnitude of HIV associated neurocognitive disorders among measurement instruments will be attributed to the difference in the ability of measurement tools to identify truly those HIV patients who have HIV associated neurocognitive disorders (sensitivity) and to exclude HIV patients who do not have HIV associated neurocognitive disorders (specificity) (33, 77–82). In addition, the lack of country-specific NP norms for the assessment of HAND would have contributed to this variation.

We did also conduct a subgroup analysis with the type of study design. The highest estimated prevalence of HAND (54.087%) was found in cohort studies (8, 21, 23, 24, 27, 29, 41, 43, 44), followed by cross-sectional studies (49.52%) (8, 10, 12–14, 17–20, 23, 25, 26, 28, 30, 31, 34–40, 45–50). The lowest estimated prevalence of HAND (44.45%) was found among case-control studies (30, 66). As noted above, the number of case-control and cohort studies is fewer in number (2 and 10 studies, respectively) compared to the number of cross-sectional studies which is 28. So the smaller number of studies included in cohort and case-control studies might be overestimated and underestimate the prevalence of HAND, respectively.

Even though we conducted a subgroup analysis with a country of origin of the study (categorized as Africa, Europe, Asia, and the USA) as a moderator variable, we found a more or less consistent average estimate of HAND; Europe (50.015%), Africa (49.566%), Asia (52.032%), and United States of America (USA) (50.407%) with the difference of prevalence estimate of HAND being insignificant.

Concerning the associated factors for HIV-associated neurocognitive disorders, our systematic review suggested that older age was among the commonly reported risk factor for HIV-associated neurocognitive disorders (8, 14, 20, 46, 49). Older age group people were 3.68 times more likely to develop HIV-associated neurocognitive disorder than younger age people. This is supported by multiple earlier studies (83). The accumulation of excessive amyloid deposits and various chronic diseases in old age could account for this (84).

Besides, depression also increases the risk of HIV-associated neurocognitive disorders as reported in three studies (14, 21, 35). The HIV-associated neurocognitive disorder was 2.87 times higher among individuals having comorbidity of depression than those not having comorbidity of depression. Multiple supportive findings for this association are reported (85–87). Pseudo-dementia associated with depression could mimic neurocognitive disorders and amplify the prevalence of neurocognitive disorders.

Moreover, advanced clinical stages of the illness (stage III and stage IV AIDS) (20, 21, 25, 49) were also associated factors for HIV-associated neurocognitive disorders. Advanced clinical stages of the illness increase the risk of HIV-associated neurocognitive disorder by 5.68.

### Difference Between Studies Incorporated in the Current Review and the Meta-Analysis Study

This meta-analysis study on HIV-associated neurocognitive disorders was subjected to a high degree of heterogeneity from the variation between 40 of the studies integrated with the final analysis. Therefore, the need for a subgroup and sensitivity analysis was mandatory. The country of origin of the study (Africa, Europe, Asia, and the USA), design of the study, and measurement tool were the parameters for subgroup analysis. However, no major difference in the pooled prevalence of HIV-associated neurocognitive disorders was observed between high-income/(Europe, Asia, and the USA) and low-to-middle income/African countries. These should be a focus of future researchers. Results of the subgroup however revealed the design of the study and measurement tool used to screen HIV-associated neurocognitive disorders were contributors to the high heterogeneity between the 40 studies integrated with the analysis. Furthermore, we did a sensitivity analysis to search for additional sources for the high degree of heterogeneity by exploring a particular study outweighing the overall estimate. However, the result showed that none of the 40 studies had outweighed the average estimated prevalence of HIV-associated neurocognitive disorders in HIV patients.

## Strength and Limitations of the Study

This systematic review and meta-analysis on HIV-associated neurocognitive disorders have several strengths. The primary strength of the study was the large number of studies included in the review, representing the worldwide spread of HAND in people with HIV. The application of subgroup analysis and sensitivity analysis to identify the source of heterogeneity was another quality of the present study. The study also used a well-designed and pre-determined search approach to lessen the reviewer's bias. The third for this study was the extraction of relevant data and assessment of the quality of 40 studies by self-determining assessors which decrease the reviewer's bias. Quality analysis of all the included studies also increases the study validity. On the other hand, the study has many limitations. First, the definition of HIV-associated neurocognitive disorder could be more explicit. In addition, the reasons for exclusions of 46 identified full texts are not explicitly mentioned in the PRISMA flowchart. The presence of a high degree of heterogeneity that influences the inference of the study outcomes was also another limitation. Moreover, biases and lack of methodological rigor could affect the validity of this finding.

## Conclusion and Recommendation

This systematic review and meta-analysis aimed to estimate the prevalence of HIV-associated neurocognitive disorder in people living with HIV AIDS and to analyze the associated factors for HIV-associated neurocognitive disorder in people with HIV AIDS. The prevalence of HIV-associated neurocognitive disorders was about 50.41%. Low level of education and older age, clinical and HIV related variables such as the advanced stage of the illness and CD4 count of 500 cells/dl or less and psychological variables such as comorbidity of depression were associated with HIV associated neurocognitive disorders. Therefore, to increase independent functioning and improve the quality of life of people with HIV/AIDS, much attention has to be given to lessening these neurocognitive disorders and adjusting the allied factors essentially through routine screening and timely intervention of HAND. Moreover, policies and procedures that integrate routine screening and timely intervention of HAND into the routine anti-retroviral therapy should be designed and implemented. Further experimental and follow-up studies with a greater sample population should be done.

## Data Availability Statement

The original contributions presented in the study are included in the article/supplementary material, further inquiries can be directed to the corresponding author.

## Author Contributions

YZ conceived the idea for the study. YZ and MN established the search approach, extracted the relevant data, accomplished the analysis, and wrote the manuscript. MN, YZ, BA, and WY did the quality assessment studies. All authors confirmed the final draft of the manuscript.

## Conflict of Interest

The authors declare that the research was conducted in the absence of any commercial or financial relationships that could be construed as a potential conflict of interest.

## Publisher's Note

All claims expressed in this article are solely those of the authors and do not necessarily represent those of their affiliated organizations, or those of the publisher, the editors and the reviewers. Any product that may be evaluated in this article, or claim that may be made by its manufacturer, is not guaranteed or endorsed by the publisher.

## Abbreviations

AIDS: Acquired Immune-Deficiency Syndrome
ANI: Asymptomatic neurocognitive impairment
ART: Anti-Retroviral Therapy
CC: case-control
CD: Cognitive decline
CS: cross-sectional
F: female
GDS: global dementia scale
HAD: HIV associated dementia
HAND: HIV associated neurocognitive disorders
IHDS: International HIV Dementia Scale
II: Intellectual impairment
M: male
MMSE: Mini-mental state exam
MND: Mild neurocognitive disorders
MoCA: Montreal Cognitive Assessment
NA: Not available
NCI: Neurocognitive impairment
SNI: Symptomatic neurocognitive impairment
UK: United Kingdom
USA: United States of America.

## References

[1] Kim AY Onofrey S Church DR. An epidemiologic update on hepatitis C infection in persons living with or at risk of HIV infection. J Infect Dis. (2013) 207:S1–S6. 10.1093/infdis/jis927 23390299 PMC3565593

[2] Chibanda D Benjamin L Weiss HA Abas M. Mental, neurological, and substance use disorders in people living with HIV/AIDS in low-and middle-income countries. JAIDS J Acquir Immune Def Syndr. (2014) 67:S54–67. 10.1097/QAI.0000000000000258 25117961

[3] Necho M Belete A Tsehay M. Depressive symptoms and their determinants in patients who are on antiretroviral therapy in the case of a low-income country, Ethiopia: a systematic review and meta-analysis. Int J Ment Health Syst. (2021) 15:1–14. 10.1186/s13033-020-00430-2 33407651 PMC7789682

[4] Ayano G Duko B Bedaso A. The prevalence of post-traumatic stress disorder among people living with HIV/AIDS: a systematic review and meta-analysis. Psychiatr Q. (2020) 91:1317–32. 10.1007/s11126-020-09849-9 32981021

[5] Necho M Belete A Getachew Y. The prevalence and factors associated with alcohol use disorder among people living with HIV/AIDS in Africa: a systematic review and meta-analysis. Subst Abuse Treat Prev Policy. (2020) 15:1–15. 10.1186/s13011-020-00301-6 32831129 PMC7444054

[6] Necho M Tsehay M Zenebe Y. Suicidal ideation, attempt, and its associated factors among HIV/AIDS patients in Africa: a systematic review and meta-analysis study. Int J Ment Health Syst. (2021) 15:1–16. 10.1186/s13033-021-00437-3 33485362 PMC7825170

[7] Mahadevan A Shankar SK Satishchandra P Ranga U Chickabasaviah YT Santosh V . Characterization of human immunodeficiency virus (HIV)-infected cells in infiltrates associated with CNS opportunistic infections in patients with HIV clade C infection. J Neuropathol Exp Neurol. (2007) 66:799–808. 10.1097/NEN.0b013e3181461d3e 17805010

[8] Troncoso FT Conterno LdO. Prevalence of neurocognitive disorders and depression in a Brazilian HIV population. Rev Soc Bras Med Trop. (2015) 48:390–8. 10.1590/0037-8682-0034-2015 26312927

[9] Chan P Brew BJ. HIV associated neurocognitive disorders in the modern antiviral treatment era: prevalence, characteristics, biomarkers, and effects of treatment. Curr HIV/AIDS Rep. (2014) 11:317–24. 10.1007/s11904-014-0221-0 24966139

[10] Nakku J Kinyanda E Hoskins S. Prevalence and factors associated with probable HIV dementia in an African population: a cross-sectional study of an HIV/AIDS clinic population. BMC Psychiatry. (2013) 13:1–7. 10.1186/1471-244X-13-126 23641703 PMC3653745

[11] Cross HM Combrinck MI Joska JA. HIV-associated neurocognitive disorders: antiretroviral regimen, central nervous system penetration effectiveness, and cognitive outcomes. South Afr Med J. (2013) 103:758–62. 10.7196/SAMJ.6677 24079630

[12] Atashili J Gaynes BN Pence BW Tayong G Kats D O'donnell JK . Prevalence, characteristics and correlates of a positive-dementia screen in patients on antiretroviral therapy in Bamenda, Cameroon: a cross-sectional study. BMC Neurol. (2013) 13:1–7. 10.1186/1471-2377-13-86 23855622 PMC3716899

[13] Lawler K Mosepele M Ratcliffe S Seloilwe E Steele K Nthobatsang R . Neurocognitive impairment among HIV-positive individuals in Botswana: a pilot study. J Int AIDS Soc. (2010) 13:1–9. 10.1186/1758-2652-13-15 20406460 PMC2876070

[14] Pinheiro C Souza L Motta J Kelbert E Souza M Martins C . Depression and diagnosis of neurocognitive impairment in HIV-positive patients. Braz J Med Biol Res. (2016) 49:e5344. 10.1590/1414-431x20165344 27626305 PMC5030831

[15] Robbins RN Scott TM Gouse H Marcotte TD Rourke SB. Screening for HIV-associated neurocognitive disorders: sensitivity and specificity. Curr Top Behav Neurosci. (2019) 50:429–78. 10.1007/7854_2019_117 32677005

[16] Habib AG Yakasai AM Owolabi LF Ibrahim A Habib ZG Gudaji M . Neurocognitive impairment in HIV-1-infected adults in Sub-Saharan Africa: a systematic review and meta-analysis. Int J Infect Dis. (2013) 17:e820–e31. 10.1016/j.ijid.2013.06.011 23953699

[17] Achappa B Priyadarshni S Madi D Bhaskaran U Ramapuram JT Rao S . Neurocognitive dysfunction among HIV positive patients using international HIV dementia scale. Asian J Med Sci. (2014) 5:61–4. 10.3126/ajms.v5i4.8724

[18] Araya T Abebaw D Belete A Derajew H Umer H. Prevalence and factors associated with neuro cognitive disorders among HIV-positive patients in Ethiopia: a hospital-based cross-sectional study. Ethiopian J Health Dev. (2020) 34:22–9.

[19] Awori V Mativo P Yonga G Shah R. The association between asymptomatic and mild neurocognitive impairment and adherence to antiretroviral therapy among people living with human immunodeficiency virus. South Afr J HIV Med. (2018) 19:674. 10.4102/sajhivmed.v19i1.674 29707383 PMC5913780

[20] Belete T Medfu G Yemiyamrew E. Prevalence of HIV asssociated neurocognitive deficit among HIV positive people in Ethiopia: A cross sectional study at ayder referral hospital. Ethiopian J Health Sci. (2017) 27:67–76. 10.4314/ejhs.v27i1.9 28458492 PMC5390230

[21] Bonnet F Amieva H Marquant F Bernard C Bruyand M Dauchy F-A . Cognitive disorders in HIV-infected patients: are they HIV-related? Aids. (2013) 27:391–400. 10.1097/QAD.0b013e32835b1019 23079813

[22] Carlson RD Rolfes MA Birkenkamp KE Nakasujja N Rajasingham R Meya DB . Predictors of neurocognitive outcomes on antiretroviral therapy after cryptococcal meningitis: a prospective cohort study. Metab Brain Dis. (2014) 29:269–79. 10.1007/s11011-013-9476-1 24399496 PMC4033836

[23] Chan LG Kandiah N Chua A. HIV-associated neurocognitive disorders (HAND) in a South Asian population-contextual application of the 2007 criteria. BMJ Open. (2012) 2:662. 10.1136/bmjopen-2011-000662 22331389 PMC3282293

[24] Cysique LA Letendre SL Ake C Jin H Franklin DR Gupta S . Incidence and nature of cognitive decline over one year among HIV-infected former plasma donors in China. AIDS (London, England). (2010) 24:983. 10.1097/QAD.0b013e32833336c8 20299964 PMC2898923

[25] Debalkie Animut M Sorrie MB Birhanu YW Teshale MY. High prevalence of neurocognitive disorders observed among adult people living with HIV/AIDS in Southern Ethiopia: A cross-sectional study. PLoS ONE. (2019) 14:e0204636. 10.1371/journal.pone.0204636 30883557 PMC6422272

[26] Elham M-T Vahid N Seyed ASA Omid D Cossarizza A Mussini C . Prevalence of HIV-associated Neurocognitive Disorder (HAND) and its subgroups among HIV-positive persons on anti-retroviral therapy in Iran. Psihologija. (2020) 53:115–27. 10.2298/PSI190414001M

[27] Focà E Magro P Motta D Compostella S Casari S Bonito A . Screening for neurocognitive impairment in HIV-infected individuals at first contact after HIV diagnosis: the experience of a large clinical center in Northern Italy. Int J Mol Sci. (2016) 17:434. 10.3390/ijms17040434 27023519 PMC4848890

[28] Haddow L Cpsychoil AA Cartledge J Manji H Benn P Gilson R. Routine detection and management of neurocognitive impairment in HIV-positive patients in a UK centre. Int J STD AIDS. (2013) 24:217–9. 10.1177/0956462412472452 23535355 PMC4138002

[29] Harezlak J Buchthal S Taylor M Schifitto G Zhong J Daar E . Persistence of hiv– associated cognitive impairment, inflammation and neuronal injury in era of highly active antiretroviral treatment. AIDS (London, England). (2011) 25:625. 10.1097/QAD.0b013e3283427da7 21297425 PMC4326227

[30] Kabuba N Menon JA Franklin DR Heaton RK Hestad KA. Use of western neuropsychological test battery in detecting HIV-associated neurocognitive disorders (HAND) in Zambia. AIDS Behav. (2017) 21:1717–27. 10.1007/s10461-016-1443-5 27278547 PMC5145764

[31] Kelly CM van Oosterhout JJ Ngwalo C Stewart RC Benjamin L Robertson KR . HIV associated neurocognitive disorders (HAND) in Malawian adults and effect on adherence to combination anti-retroviral therapy: a cross sectional study. PLoS ONE. (2014) 9:e98962. 10.1371/journal.pone.0098962 24915530 PMC4051684

[32] Lindayani L Sudrajat DA Melnawati C Anggarini D. Prevalence of neurocognitive impairment and its associated factors among patients with HIV in Indonesia. Br J Neurosci Nurs. (2020) 16:258–64. 10.12968/bjnn.2020.16.6.258

[33] Marin-Webb V Jessen H Kopp U Jessen AB Hahn K. Validation of the international HIV dementia scale as a screening tool for HIV-associated neurocognitive disorders in a German-speaking HIV outpatient clinic. PLoS ONE. (2016) 11:e0168225. 10.1371/journal.pone.0168225 27992497 PMC5167352

[34] McNamara PH Coen R Redmond J Doherty CP Bergin C editors. A High Prevalence Rate of a positive Screen for Cognitive Impairment in Patients With Human Immunodeficiency Virus Attending an Irish Clinic. Open Forum Infectious Diseases: Oxford: Oxford University Press US (2017). 10.1093/ofid/ofw242 PMC5414021 28480240

[35] Mugendi A Kubo M Nyamu D Mwaniki L Wahome S Haberer J. Prevalence and correlates of neurocognitive disorders among HIV patients on antiretroviral therapy at a Kenyan hospital. Neurol Res Int. (2019) 2019:5173289. 10.1155/2019/5173289 31781391 PMC6875169

[36] Muniyandi K Venkatesan J Arutselvi T Jayaseelan V. Study to assess the prevalence, nature and extent of cognitive impairment in people living with AIDS. Indian J Psychiatry. (2012) 54:149. 10.4103/0019-5545.99534 22988322 PMC3440909

[37] Nakasujja N Allebeck P Agren H Musisi S Katabira E. Cognitive dysfunction among HIV positive and HIV negative patients with psychosis in Uganda. PLoS ONE. (2012) 7:e44415. 10.1371/journal.pone.0044415 22970214 PMC3435287

[38] Nyamayaro P Gouse H Hakim J Robbins RN Chibanda D. Neurocognitive impairment in treatment-experienced adults living with HIV attending primary care clinics in Zimbabwe. BMC Infect Dis. (2020) 20:1–10. 10.1186/s12879-020-05090-8 32471350 PMC7257139

[39] Pascal M Gaspard T Philomène N-K Emmanuel Y Avilah A-WP Hortense H. Determinants of neurocognitive impairment in HIV in a cohort of patients on antiretroviral therapy followed in Bangui (Central African Republic). Neurosci. Med. (2016) 7:1. 10.4236/nm.2016.71001

[40] Robertson K Bayon C Molina J-M McNamara P Resch C Muñoz-Moreno JA . Screening for neurocognitive impairment, depression, and anxiety in HIV-infected patients in Western Europe and Canada. AIDS Care. (2014) 26:1555–61. 10.1080/09540121.2014.936813 25029599 PMC4193282

[41] Robertson KR Smurzynski M Parsons TD Wu K Bosch RJ Wu J . The prevalence and incidence of neurocognitive impairment in the HAART era. Aids. (2007) 21:1915–21. 10.1097/QAD.0b013e32828e4e27 17721099

[42] Sacktor N Skolasky RL Seaberg E Munro C Becker JT Martin E . Prevalence of HIV-associated neurocognitive disorders in the Multicenter AIDS Cohort Study. Neurology. (2016) 86:334–40. 10.1212/WNL.0000000000002277 26718568 PMC4776086

[43] Saini S Barar KV. Assessment of neurocognitive functions in HIV/AIDS patients on HAART using the international HIV dementia scale. Int J Nutr Pharmacol Neurol Dis. (2014) 4:252. 10.4103/2231-0738.139408

[44] Simioni S Cavassini M Annoni J-M Abraham AR Bourquin I Schiffer V . Cognitive dysfunction in HIV patients despite long-standing suppression of viremia. Aids. (2010) 24:1243–50. 10.1097/QAD.0b013e3283354a7b 19996937

[45] Sunmonu TA Sellner J Ogunrin OA Imarhiagbe FA Komolafe MA Afolabi OT . Intellectual impairment in patients with newly diagnosed HIV infection in southwestern Nigeria. Biomed Res Int. (2015) 2015:185891. 10.1155/2015/185891 26295033 PMC4532809

[46] Tsegaw M Andargie G Alem G Tareke M. Screening HIV-associated neurocognitive disorders (HAND) among HIV positive patients attending antiretroviral therapy in South Wollo, Ethiopia. J Psychiatr Res. (2017) 85:37–41. 10.1016/j.jpsychires.2016.10.016 27821271

[47] Yakasai AM Gudaji MI Muhammad H Ibrahim A Owolabi LF Ibrahim DA . Prevalence and correlates of HIV-associated neurocognitive disorders (HAND) in Northwestern Nigeria. Neurol Res Int. (2015) 2015:486960. 10.1155/2015/486960 26347017 PMC4546766

[48] Yideg Yitbarek G Mossie Ayana A Bariso Gare M Garedew Woldeamanuel G. Prevalence of cognitive impairment and its predictors among HIV/AIDS patients on antiretroviral therapy in Jimma University medical center, Southwest Ethiopia. Psychiatry J. (2019) 2019:8306823. 10.1155/2019/8306823 31001550 PMC6436374

[49] Yitbarek GY Ayehu GW Ayele BA Bayih WA Gebremariam AD Tiruneh SA. Cognitive impairment and its associated factors among HIV/AIDS patients on anti retro-viral therapy in Sub-Saharan Africa: systematic review and meta-analysis. Neurol Psychiatry Brain Res. (2020) 38:83–91. 10.1016/j.npbr.2020.11.002

[50] Yusuf AJ Hassan A Mamman AI Muktar HM Suleiman AM Baiyewu O. Prevalence of HIV-associated neurocognitive disorder (HAND) among patients attending a tertiary health facility in Northern Nigeria. J Int Assoc Providers AIDS Care. (2017) 16:48–55. 10.1177/2325957414553839 25331222 PMC4404171

[51] Van Wijk C. Screening for HIV-associated neurocognitive disorders (HANDs) in South Africa: A caution against uncritical use of comparative data from other developing countries. South Afr J HIV Med. (2013) 14:97. 10.4102/sajhivmed.v14i1.97

[52] Joska JA Dreyer AJ Nightingale S Combrinck MI De Jager CA. Prevalence of HIV-1 Infection in an elderly rural population and associations with neurocognitive impairment. Aids. (2019) 33:1765–71. 10.1097/QAD.0000000000002257 31361273

[53] Patel V Mungwira R Tarumbiswa T Heikinheimo T Van Oosterhout J. High prevalence of suspected HIV-associated dementia in adult Malawian HIV patients. Int J STD AIDS. (2010) 21:356–8. 10.1258/ijsa.2010.009554 20498107

[54] Rao VR Ruiz AP Prasad VR. Viral and cellular factors underlying neuropathogenesis in HIV associated neurocognitive disorders (HAND). AIDS Res Ther. (2014) 11:1–15. 10.1186/1742-6405-11-13 24894206 PMC4043700

[55] Mossie TB Tegegne MT. HIV dementia among HIV positive people at Debre markos hospital, Northwest Ethiopia. Am J Psychol Neurosci. (2014) 2:18–24. 10.11648/j.ajpn.20140202.11

[56] Moher D Shamseer L Clarke M Ghersi D Liberati A Petticrew M . Preferred reporting items for systematic review and meta-analysis protocols (PRISMA-P) 2015 statement. Syst Rev. (2015) 4:1. 10.1186/2046-4053-4-1 25554246 PMC4320440

[57] Barendregt JJ Doi SA. MetaXL user guide. Version. (2016) 4:2011–6.

[58] Stang A. Critical evaluation of the Newcastle-Ottawa scale for the assessment of the quality of nonrandomized studies in meta-analyses. Eur J Epidemiol. (2010) 25:603–5. 10.1007/s10654-010-9491-z 20652370

[59] Doi SA Thalib L. A quality-effects model for meta-analysis. Epidemiology. (2008) 19:94–100. 10.1097/EDE.0b013e31815c24e7 18090860

[60] Nyaga VN Arbyn M Aerts M. Metaprop: a Stata command to perform meta-analysis of binomial data. Arch Public Health. (2014) 72:39. 10.1186/2049-3258-72-39 25810908 PMC4373114

[61] Higgins JP Thompson SG. Quantifying heterogeneity in a meta-analysis. Stat Med. (2002) 21:1539–58. 10.1002/sim.1186 12111919

[62] Liu JL. The role of the funnel plot in detecting publication and related biases in meta-analysis. Evid Based Dent. (2011) 12:121. 10.1038/sj.ebd.6400831 22193659

[63] Yechoor N Towe SL Robertson KR Westreich D Nakasujja N Meade CS. Utility of a brief computerized battery to assess HIV-associated neurocognitive impairment in a resource-limited setting. J Neurovirol. (2016) 22:808–15. 10.1007/s13365-016-0456-1 27245592 PMC5130618

[64] Fasel D Kunze U Elzi L Werder V Niepmann S Monsch AU . A short tool to screen HIV-infected patients for mild neurocognitive disorders - a pilot study. BMC Psychol. (2014) 2:5004. 10.1186/2050-7283-2-21 25815192 PMC4363199

[65] Oshinaike OO Akinbami AA Ojo OO Ojini IF Okubdejo UN Danesi AM. Comparison of the minimental state examination scale and the international HIV dementia scale in assessing cognitive function in nigerian HIV patients on antiretroviral therapy. AIDS Res Treat. (2012) 2012:581531. 10.1155/2012/581531 23050130 PMC3463159

[66] Gouse H Masson CJ Henry M Marcotte TD London L Kew G . Assessing HIV provider knowledge, screening practices, and training needs for HIV-associated neurocognitive disorders. A short report. Aids Care. (2021). 33:468–72. 32138523 10.1080/09540121.2020.1736256 PMC7483165

[67] Ayano G Betts K Maravilla JC Alati R. A systematic review and meta-analysis of the risk of disruptive behavioral disorders in the offspring of parents with severe psychiatric disorders. Child Psychiatry Hum Dev. (2021) 52:77–95. 10.1007/s10578-020-00989-4 32291561

[68] Hobson P Lewis A Nair H Wong S Kumwenda M. How common are neurocognitive disorders in patients with chronic kidney disease and diabetes? Results from a cross-sectional study in a community cohort of patients in North Wales, UK. BMJ Open. (2018) 8:e023520. 10.1136/bmjopen-2018-023520 30518585 PMC6286490

[69] Vas CJ Pinto C Panikker D Noronha S Deshpande N Kulkarni L . Prevalence of dementia in an urban Indian population. Int Psychogeriatr. (2001) 13:439. 10.1017/S1041610201007852 12003250

[70] Gurvit H Emre M Tinaz S Bilgic B Hanagasi H Sahin H . The prevalence of dementia in an urban Turkish population. Am J Alzheimers Dis Other Dement. (2008) 23:67–76. 10.1177/1533317507310570 18276959 PMC10846186

[71] Engedal K Haugen PK. The prevalence of dementia in a sample of elderly Norwegians. Int J Geriatr Psychiatry. (1993) 8:565–70. 10.1002/gps.930080706

[72] Ueda K Kawano H Hasuo Y Fujishima M. Prevalence and etiology of dementia in a Japanese community. Stroke. (1992) 23:798–803. 10.1161/01.STR.23.6.798 1595095

[73] Tan IL Smith BR von Geldern G Mateen FJ McArthur JC. HIV-associated opportunistic infections of the CNS. Lancet Neurol. (2012) 11:605–17. 10.1016/S1474-4422(12)70098-4 22710754

[74] Bowen LN Smith B Reich D Quezado M Nath A. HIV-associated opportunistic CNS infections: pathophysiology, diagnosis and treatment. Nat Rev Neurol. (2016) 12:662. 10.1038/nrneurol.2016.149 27786246

[75] Anthony I Ramage S Carnie F Simmonds P Bell J. Influence of HAART on HIV-related CNS disease and neuroinflammation. J Neuropathol Exp Neurol. (2005) 64:529–36. 10.1093/jnen/64.6.529 15977645

[76] Wei J Hou J Su B Jiang T Guo C Wang W . The Prevalence of Frascati-Criteria-Based HIV-Associated Neurocognitive Disorder (HAND) in HIV-Infected Adults: A Systematic Review and Meta-Analysis. Front Neurol. (2020) 11:1613. 10.3389/fneur.2020.581346 33335509 PMC7736554

[77] Phillips NJ Thomas KG Myer L Sacktor N Zar HJ Stein DJ . Screening for HIV-associated neurocognitive disorders in perinatally infected adolescents: youth-International HIV Dementia Scale validation. Aids. (2019) 33:815–24. 10.1097/QAD.0000000000002144 30649059

[78] López E Steiner AJ Smith K Thaler NS Hardy DJ Levine AJ . Diagnostic utility of the HIV dementia scale and the international HIV dementia scale in screening for HIV-associated neurocognitive disorders among Spanish-speaking adults. Appl Neuropsychol. (2017) 24:512–21. 10.1080/23279095.2016.1214835 27712132 PMC5938065

[79] Bloch M Kamminga J Jayewardene A Bailey M Carberry A Vincent T . A screening strategy for HIV-associated neurocognitive disorders that accurately identifies patients requiring neurological review. Clin Infect Dis. (2016) 63:687–93. 10.1093/cid/ciw399 27325690 PMC4981762

[80] Joska J Witten J Thomas K Robertson C Casson-Crook M Roosa H . A comparison of five brief screening tools for HIV-associated neurocognitive disorders in the USA and South Africa. AIDS Behav. (2016) 20:1621–31. 10.1007/s10461-016-1316-y 26860536 PMC5771655

[81] Rosca EC Albarqouni L Simu M. Montreal cognitive assessment (MoCA) for HIV-associated neurocognitive disorders. Neuropsychol Rev. (2019) 29:313–27. 10.1007/s11065-019-09412-9 31440882

[82] Kim WJ Ku NS Lee Y-J Ahn JY Kim SB Ahn H-W . Utility of the montreal cognitive assessment (MoCA) and its subset in HIV-associated neurocognitive disorder (HAND) screening. J Psychosom Res. (2016) 80:53–7. 10.1016/j.jpsychores.2015.11.006 26721548

[83] Mecocci P Boccardi V. The impact of aging in dementia: it is time to refocus attention on the main risk factor of dementia. Ageing Res Rev. (2020) 2020:101210. 10.1016/j.arr.2020.101210 33186671

[84] Abbott A. Dementia: a problem for our age. Nature. (2011) 475:S2–S4. 10.1038/475S2a 21760579

[85] Jorm AF. History of depression as a risk factor for dementia: an updated review. Austr N Z J Psychiatry. (2001) 35:776–81. 10.1046/j.1440-1614.2001.00967.x 11990888

[86] Cantón-Habas V Rich-Ruiz M Romero-Saldaña M Carrera-González MdP. Depression as a risk factor for dementia and alzheimer's disease. Biomedicines. (2020) 8:457. 10.3390/biomedicines8110457 33126696 PMC7693751

[87] Helvik A-S Barca ML Bergh S Šaltyte-Benth J Kirkevold Ø Borza T. The course of depressive symptoms with decline in cognitive function-a longitudinal study of older adults receiving in-home care at baseline. BMC Geriatr. (2019) 19:1–14. 10.1186/s12877-019-1226-8 31443638 PMC6708209

